# Comparison of the therapeutic effects of different pneumoperitoneum pressures on laparoscopic transabdominal preperitoneal hernia repair: a randomized controlled trail

**DOI:** 10.1590/acb399824

**Published:** 2024-12-06

**Authors:** Jie Yao, Shichen Qin, Guang Yang

**Affiliations:** 1 Haimen People’s Hospital affiliated to Nantong University – Department of General Surgery – Nantong – China.; 2 Southeast University – School of Medicine – Nanjing – China.; 3 Haimen District People’s Hospital – Department of General Surgery – Nantong – China.

**Keywords:** Hernia, Laparoscopy, Pneumoperitoneum

## Abstract

**Purpose:**

To compare the indicators, postoperative pneumoretroperitoneum-related complications, and postoperative recovery of laparoscopic preperitoneal inguinal hernia repair under different CO_2_ pneumoperitoneum pressures.

**Methods:**

The total of 187 adult patients with primary inguinal hernia who successfully underwent transabdominal preperitoneal prosthesis (TAPP) from September 2021 to September 2023 in the Department of General Surgery, Haimen People’s Hospital affiliated to Nantong University, were collected. These patients were randomly divided into low abdominal pressure group (group A: pneumoperitoneum pressure = 8 mmHg), sub-low abdominal pressure group (group B: pneumoperitoneum pressure = 10 mmHg), moderate abdominal pressure group (group C: pneumoperitoneum pressure = 12 mmHg), and standard pressure group (group D: pneumoperitoneum pressure = 14 mmHg), with 40 patients each.

**Results:**

The operation time in group C (43.90 ± 9.75) was significantly lower than group A (51.98 ± 12.65, *p* 0.001), group B (46.70 ± 10.59, *p* 0.001), and was higher than that in group D without significant statistical differences (38.15 ± 7.98, *P* = 0.05). The peritoneal suturing time in group C (5.03 ± 1.07) was significantly higher than group A (4.23 ± 0.70, *p* 0.001), group B (4.55 ± 0.85, *p* = 0.03), and was significantly lower than that in group D (6.95 ± 1.96, *p* 0.001).

**Conclusion:**

Selecting sub-low abdominal pressure (12 mmHg) can help to have a shorter operation time, with less blood loss, and it did not add pneumoretroperitoneum-related complications. Changing the pneumoperitonium pressure during different phases of the surgery is also an optimal option.

## Introduction

The transabdominal preperitoneal hernia repair (TAPP) has achieved widespread application, and its efficacy and safety have been widely recognized in the standard surgical procedures for treating various types of inguinal hernias^1^^-^ 3. Studies have shown that, compared to traditional open hernia surgery, TAPP offers advantages such as reduced postoperative pain, shorter hospital stays, lower complication rates, and faster postoperative recoveries^4^^-^ 6. Moreover, TAPP has greater surgical workspace, better exposure, simpler operations, less trauma, and shorter operation times, facilitating the observation of contralateral occult hernias and their contents^7^^-^ 9. 

Conventional TAPP requires the establishment of pneumoperitoneum and maintaining a CO_2_ pneumoperitoneal pressure of 13 to 15 mmHg to ensure a clear surgical field and an operable environment. However, complications related to pneumoperitoneum arise, and the damage and complications caused by it have been a concern^10^^,^ 11. Pneumoperitoneum may affect physiological functions such as respiration and circulation, especially in elderly patients, in which it can lead to serious complications such as acidosis, respiratory and cardiac arrest, and venous CO_2_ embolism^12^^,^ 13. Studies have shown that about 10 to 15% of patients undergoing conventional laparoscopic surgery experience increased heart rate^14^, increased airway pressure^14^, elevated blood pressure^15^, decreased cardiac output^16^, decreased oxygen saturation^17^, and others^14^^,^ 18 ^,^ 19. The reasons may be related to the high pneumoperitoneal pressure and the rapid diffusion of CO_2_ into the circulation, leading to hypercapnia^20^. Other complications related to pneumoperitoneum include hypothermia, acid-base imbalance, and effects on bowel function. In theory, a higher pneumoperitoneal pressure provides a larger operational space with less intraoperative dissection, potentially reducing intraoperative bleeding, postoperative pain, and length of hospital stay. However, this is accompanied by an increase in the risk of complications. Among the various methods to prevent these complications, reducing pneumoperitoneal pressure is a simple and effective approach^21^.

Therefore, selecting an appropriate pneumoperitoneal pressure that is both safe and effective for TAPP surgery is crucial for reducing the occurrence of complications and ensuring safe and effective patient outcomes. We conducted a prospective clinical trial to investigate the application of different pneumoperitoneal pressures in TAPP surgery. The aims were to compare and observe the effects of different pneumoperitoneal pressures on intraoperative indicators, postoperative complications, and recovery outcomes of TAPP surgery. This approach aimed to identify a safe and effective pneumoperitoneal pressure value that provides optimal surgical conditions while minimizing the occurrence of complications.

## Methods

### Clinical data collection and inclusion/exclusion criteria

Prospectively collected 187 adult patients with primary inguinal hernia who successfully underwent TAPP in our department from September 2021 to September 2023 in the Department of General Surgery, Haimen People’s Hospital affiliated to Nantong University. Inclusion criteria: 18–70 years old, appropriate body mass index (BMI), unilateral primary inguinal hernia, American Society of Anesthesiologists (ASA) physical status classification of I-II22, good patient compliance, no psychological illness, and able to tolerate surgery. Exclusion criteria: scrotal hernia, irreducible hernia, femoral hernia, incarcerated hernia, history of complex lower abdominal surgery, intestinal necrosis requiring partial resection of the intestine in the hernia, intraoperative discovery of bilateral hernias, establishment of pneumoperitoneum time < 40 min or > 90 min, conversion to open surgery for various reasons, and severe obesity or BMI > 37.5.

This study is a prospective randomized controlled study and has been approved by the hospital ethics committee (approval N^o^. 2021-KY17).

The patients were randomly divided into low pneumoperitoneum pressure group (group A: pneumoperitoneum pressure = 8 mmHg), sub-low pneumoperitoneum pressure group (group B: pneumoperitoneum pressure = 10 mmHg), moderate pneumoperitoneum pressure group (group C: pneumoperitoneum pressure = 12 mmHg), and standard pneumoperitoneum pressure group (group D: pneumoperitoneum pressure = 14 mmHg), with 40 patients in each group. The grouping was proceeded by a nurse according to the random table, and the nurse was blinded to this study protocol. All patients were randomly numbered according to a random number table, and then the number was marked on the ball which represented the patient. After all patients were marked on the balls, a nurse randomly selected one ball (one patient) and assigned him/her to group A, one to group B, one to group C, one to group D, and so on. Until all the patients were assigned to groups, the grouping was completed.

**Figure 1:**
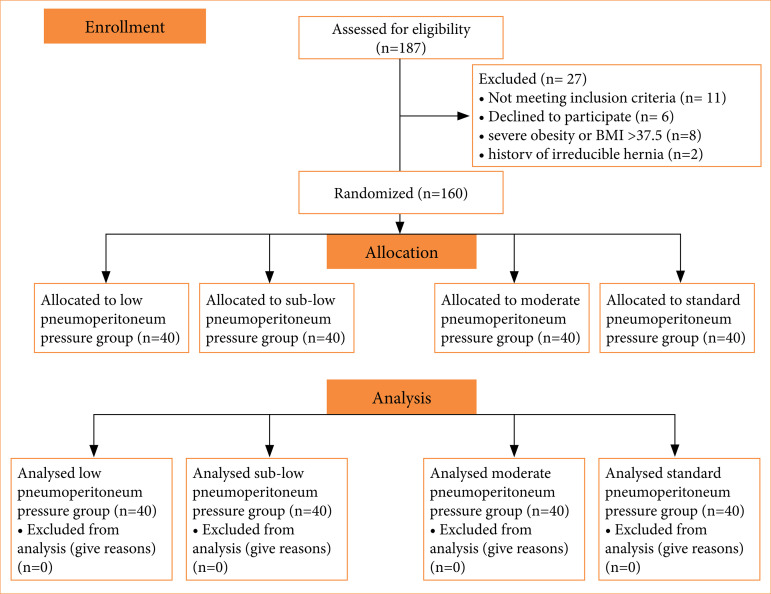
The consort diagram of the patients included.

Source: Elaborated by the authors.

Preoperatively, the patients and their families were fully informed of the study details and signed informed consent forms. Finally, 160 patients were included in this study (Fig. 1).

### Observation indicators

Main outcomes included operation time (preperitoneal suturing time), peritoneal suturing time, intraoperative blood loss, and postoperative inguinal pain greater than grade 4. Secondary outcomes included postoperative subcutaneous emphysema (inguinal region), scrotal emphysema, abdominal wall tension after peritoneal suturing, residual small holes in the peritoneum, postoperative time to getting out of bed, and postoperative hospital stay.

Among them, abdominal wall tension and residual small holes in the peritoneum were observed and recorded intraoperatively. Postoperative inguinal pain was assessed by a two-day follow-up after surgery using the commonly used numerical rating scale for postoperative patient pain assessment^23^. Postoperative pain is a subjective experience and can be directly communicated with the patient if they are conscious. Zero represents no pain, 10 represents unbearable pain. The pain is divided into 0–10 different levels. Among them, 1–3 points belong to mild pain, 4–6 points are moderate pain, and 7–10 points are severe pain.

### Anesthesia

All patients included in the study were given intravenous combined with inhaled anesthesia. Thirty minutes before anesthesia induction, intramuscular injection of phenobarbital sodium 0.1 g and atropine 0.5 mg was given. Anesthesia induction was performed using midazolam 0.05 mg/kg, cisatracurium 0.2 mg/kg, propofol 2 mg/kg, and sufentanil 0.5 μg/kg. After tracheal intubation, mechanical ventilation with positive end-expiratory pressure (3 mmHg) was performed for 15 minutes (pure oxygen inhalation at 100%, respiratory rate of 12 breaths/min, tidal volume set to 10 mL/kg, respiratory ratio of 1:2). According to the end-tidal CO_2_ partial pressure, the minute ventilation volume was adjusted to maintain the end-tidal CO_2_ partial pressure < 50 mmHg. The patient’s spontaneous breathing recovered, and consciousness returned when the patient was extubated after surgery.

### Surgical method

The patient was placed in a head-low buttocks position, and all surgical procedures were performed by a single surgeon following the guidelines for laparoscopic inguinal hernia surgery in China in 2017.

### Sample size

The sample size in this study referred to the reference by Bacchetti^24^. The author suggested that effect sizes and proposed sample sizes can be arbitrary in human studies. To better complete this study, we set the study size to be at least 40 in each group.

### Statistical processing

Statistical analysis was performed using GraphPad Prism 8 software. Continuous variables (age, BMI, operation time [preperitoneal suturing time, peritoneal suturing time], blood loss, postoperative time to getting out of bed, postoperative hospital stay) were expressed as means ± standard deviations ([x ± s]). Categorical variables (gender, anesthesia ASA classification, abdominal wall tension after peritoneal suturing, residual small holes in the peritoneum, postoperative inguinal pain greater than grade 4) were analyzed using the χ^2^ test or the corrected χ^2^ test or Fisher’s exact test. *p* < 0.05 was considered statistically significant.

## Results

### Comparisons of demographic and baseline data among groups

Seven individuals were excluded due to lack of data on admitted patients, 20 patients changed the intraoperative surgery for various reasons, such as from laparoscopic preperitoneal inguinal hernia repair to open surgery, combination of other complex hernias or combination of intestinal necrosis. A total of 160 patients was included and grouped into the following four groups, A, B, C, and D. There were no significant differences in gender, age, BMI, and preoperative ASA scores among the four groups (Table 1). Therefore, the four groups of patients were comparable.

**Table 1:** Comparisons of demographic and surgical data among groups.

Group	Gender		Age (year)		ASA		Operation time (min)	Peritoneal suturing time (min)	Amount of bleeding (mL)
	Male	Female			I	II			
A	35	5	57.73 ± 9.38	25.05 ± 1.18	32	8	51.98 ± 12.65	4.23 ± 0.70	26.55 ± 7.53
B	34	6	57.80 ± 9.74	25.13 ± 1.22	30	10	46.70 ± 10.59	4.55 ± 0.85	25.08 ± 5.90
C	35	5	59.68 ± 8.74	25.39 ± 1.23	34	6	43.90 ± 9.75	5.03 ± 1.07	22.05 ± 6.24
D	34	6	59.13 ± 9.59	25.38 ± 1.20	34	6	38.15 ± 7.98	6.95 ± 1.96	11.38 ± 4.56
F/χ^2^	0.21		0.43	0.80	0.11		63.46	38.42	49.65
*p*-value	0.98		0.73	0.50	0.99		< 0.0001	< 0.0001	< 0.0001

BMI: body mass index; ASA: American Society of Anesthesiologists. Source: Elaborated by the authors.

There were significant differences in operation time, peritoneal suturing time, and blood loss among the four groups (Table 1). The operation time in group C (43.90 ± 9.75) was significantly lower than in group A (51.98 ± 12.65, *p* < 0.001), group B (46.70 ± 10.59, *p* < 0.001), and was higher than in group D without significant statistical differences (38.15 ± 7.98, *p* = 0.05). The peritoneal suturing time in group C (5.03 ± 1.07) was significantly higher than in group A (4.23 ± 0.70, *p* < 0.001), group B (4.55 ± 0.85, *p* = 0.03), and was significantly lower than in group D (6.95 ± 1.96, *p* < 0.001). The intraoperative blood loss in group C (22.05 ±6 .24) was significantly less than in group A (26.55 ± 7.53, *p* < 0.001), group B (25.08 ± 5.90, *p* < 0.001), and was significantly more than in group D (11.38 ± 4.56, *p* < 0.001).

Incidence of postoperative pneumoretroperitoneum-related complications in each group

There was no significant difference in the incidence of postoperative pneumoretroperitoneum-related complications among the groups (Table 2). However, compared with group D, the incidence of subcutaneous emphysema was significantly lower in groups A, B, and C, with *p*-values of 0.01, 0.03, and 0.04, respectively. There was no difference in other parameters between the two groups.

**Table 2:** Comparison of postoperative pneumoretroperitoneum-related complications among groups.

Group	Subcutaneous emphysema (n/%)	Scrotal emphysema (n/%)	Abdominal tension (n/%)	Abdominal residual small hole (n/%)	Postoperative pain > Level 4 (n/%)	Time to first postoperative mobilization (h)	Postoperative hospital stay (d)	Postoperative SPO_2_ (%)
A	1 (2.6)	1 (2.6)	1 (2.6)	0 (0)	3 (7.5)	4.55 ± 1.06	2.23 ± 0.53	98.95 ± 0.81
B	2 (5)	2 (5)	2 (5)	2 (5)	2 (5)	4.63 ± 1.17	2.28 ± 0.55	98.87 ± 0.83
C	5 (12.5)	4 (10)	3 (7.5)	3 (7.5)	2 (5)	4.53 ± 1.01	2.29 ± 0.53	98.47 ± 0.73
D	10 (25)	6/34 (15)	4 (10)	4 (10)	1 (2.6)	4.98 ± 1.29	2.30 ± 0.60	98.42 ± 0.95
F/χ^2^	12.27	4.94	2.13	4.12	1.05	1.34	0.18	1.87
*p*-value	0.01	0.18	0.5452	0.2387	0.79	0.26	0.91	0.28

Source: Elaborated by the authors.

## Discussion

As a commonly used and highly regarded surgical technique for treating various types of inguinal hernias, the effectiveness and safety of TAPP have been extensively validated, establishing its standard status in hernia repair surgery^1^^-^ 3 ^,^ 25. As stated in previous reports, pneumoperitoneum can affect respiratory and circulatory functions, particularly in elderly patients, and may lead to severe complications such as acidosis, respiratory and cardiac arrest, and venous CO_2_ embolism^24^^-^ 27.

We conducted a prospective clinical study exploring the use of different pneumoperitoneum pressures in TAPP surgery, aiming to determine a safe and effective pressure range that optimizes surgical conditions while minimizing complications. Our study found that, under different pneumoperitoneum pressures (8, 10, 12, and 14 mmHg), there were significant differences in operation time, peritoneal suturing time, and blood loss. The higher the pneumoperitoneum pressure, the shorter the operation time, the less blood loss. However, the highest pneumoperitoneum pressure in group D showed more subcutaneous emphysema, which should be considered in the clinical practice.

A recent study found that there were no significant differences in blood loss, hospital stay, and the occurrence of scrotal emphysema among the low pressure, standard pressure, and high-pressure groups, but the operation time in the low pressure group was slightly longer than that in the standard pressure and high-pressure groups^28^. However, the high-pressure group had significantly higher intra- and post-operative PaCO_2_ levels, subcutaneous emphysema, and scrotal emphysema rates compared to the low-pressure and standard pressure groups^28^. This study^28^ is consistent with our findings. We found that there were significant differences in operation time, peritoneal suturing time, and blood loss in the low abdominal pressure group, the sub-low abdominal pressure group than the medium abdominal pressure group, and the standard abdominal pressure group. We speculate that the reason is that lower pneumoperitoneum pressure results in smaller exposed surgical space and poorer visibility, making it more difficult to separate the peritoneum from the hernial sac^24^^-^ 27. Therefore, the operation is more challenging and takes longer. When suturing the peritoneum, lower pneumoperitoneum pressure results in less peritoneal tension^29^^,^ 30.

As the pneumoperitoneum pressure increases, the peritoneal tension also increases. When the peritoneal tension is low, it is easier to place a mesh patch or suture the peritoneum without residual small holes in the peritoneum, resulting in a shorter suturing time^29^^-^ 31. When the pneumoperitoneum pressure is low, there may be more blood loss due to the limited surgical space and poor exposure during the dissection of the hernial sac or surrounding tissue^30^^,^ 31. However, when the pneumoperitoneum pressure is high, the surgical space is clear with well-defined planes, allowing easy dissection in avascular areas and less bleeding^30^^-^ 32. Our study findings underscore the importance of carefully balancing pneumoperitoneum pressure to optimize surgical outcomes and patient safety.

The results of our study did not find significant differences in subcutaneous emphysema, scrotal emphysema, peritoneal tension, residual small holes in the peritoneum, postoperative pain (grade > 4), time to ambulation, and hospital stay among the low abdominal pressure group, the sub-low abdominal pressure group, the medium abdominal pressure group, and the standard abdominal pressure group. Scrotal emphysema is a specific type of subcutaneous emphysema and also a pneumoperitoneum-related complication^20^. Using appropriate pneumoperitoneum pressure is a relatively safe, simple, and effective way to reduce pneumoperitoneum-related complications^33^. In the low abdominal pressure group, we found that there were no differences in peritoneal suturing time and blood loss compared to the sub-low abdominal pressure group. Additionally, the proportion of subcutaneous emphysema was significantly lower in the low abdominal pressure and sub-low abdominal pressure groups compared to the standard abdominal pressure group. Therefore, sub-low abdominal pressure is a relatively ideal TAPP pneumoperitoneum pressure.

There are also some limitations in this study. For example, the sample size is not sufficient, and it is not possible to directly reflect the differences in the incidences of subcutaneous emphysema, scrotal emphysema, peritoneal tension, residual small holes in the peritoneum, and postoperative pain (grade > 4) among the groups. Additionally, changes in PaCO_2_ during surgery were not monitored among the different groups, so it cannot be determined whether different pneumoperitoneum pressures have an impact on PaCO_2_ levels and the risk of developing hypercapnia.

## Conclusion

In summary, pneumoperitoneum pressure at 12 mmHg may be relatively safe, it does not increase intraoperative blood loss or surgical complications, and it provides a relatively large surgical space, reducing surgical difficulty. Changing the pneumoperitonium pressure during different phases of the surgery is an optimal option. We suggest a method in which most of the surgery is conducted at a relatively high pressure, reducing it only for peritoneal suturing, to get the optimal surgery effect. However, further studies with larger sample sizes and monitoring of PaCO_2_ levels are needed to confirm these findings and to better understand the optimal pneumoperitoneum pressure for specific surgical procedures.

### Ethics approval and consent to participate

This study was proceeded in line with the Declaration of Helsinki, and approved by the ethics committee of Haimen People’s Hospital affiliated to Nantong University (approval No. 2021-KY17). The patients and their families were fully informed of the study details and signed informed consent forms.

## Data Availability

The datasets analyzed during the current study are available from the corresponding author on reasonable request.
